# Development and Psychometric Evaluation of the Hope in Medicine Scale

**DOI:** 10.32872/cpe.12001

**Published:** 2024-03-28

**Authors:** Lea Balthasar, Anne-Kathrin Bräscher, Ted J. Kaptchuk, Sarah K. Ballou, Tobias Kube

**Affiliations:** 1 Pain and Psychotherapy Research Lab, RPTU Kaiserslautern-Landau, Landau, Germany; 2 Department of Clinical Psychology, Psychotherapy, and Experimental Psychopathology, Johannes Gutenberg University of Mainz, Mainz, Germany; 3 Program in Placebo Studies, Beth Israel Deaconess Medical Center, Harvard Medical School, Boston, MA, USA; 4 Division of Gastroenterology, Beth Israel Deaconess Medical Center, Harvard Medical School, Boston, MA, USA; Philipps-University of Marburg, Marburg, Germany

**Keywords:** hope, placebo, questionnaire, self-report, allergic rhinitis

## Abstract

**Background:**

Hope is an integral, multi-dimensional part of seeking medical treatment. The aim of this study was to develop a self-report scale, the Hope in Medicine (HIM) scale, to measure different modes of hoping in relation to the course of symptoms, the effects of treatment, and supporting medical research.

**Method:**

We examined the psychometric properties of the scale in a sample of 74 allergic rhinitis patients participating in a 2-week randomized-controlled trial comparing open-label placebos (OLP) with treatment as usual (TAU).

**Results:**

The HIM scale had a Cronbach’s α of .78. An exploratory factor analysis revealed four factors: realistic hope (i.e., hoping for specific positive outcomes such as improvement in symptoms), transcendent hope (i.e., non-directed hoping that things will turn out positively), utopian hope (i.e., hoping to contribute to greater knowledge), and technoscience hope (i.e., hoping for scientific breakthroughs). Speaking to the convergent validity of the scale, realistic hope was moderately related to treatment expectancies (r = .54); transcendent hope was related to optimism (r = .50), treatment expectancies (r = .37), self-efficacy (r = .36), and inversely correlated with pessimism (r = -.43). Hope subscales predicted neither course of symptoms nor impairment.

**Conclusion:**

The HIM scale is a questionnaire with adequate internal consistency allowing to assess four modes of hoping. Preliminary results for its convergent validity are promising. Yet, further validation is needed.

## Highlights

- The HIM scale was developed to assess hope specifically in relation to treatment.
- The HIM scale allows to assess several modes of hoping.
- The study shows promising results concerning internal consistency and convergent validity of the HIM scale.

Hope has been the subject of scrutiny across academic disciplines, including philosophy, psychology, and medicine. It is an integral aspect of human life (Webb, 2007) and is often present in everyday life (e.g., hoping for a promotion or a general hopefulness for a bright future). When facing a serious illness, patients may hope for a remission of their symptoms and/or successful treatment outcomes. Even terminally ill, patients often maintain hope, e.g., hoping to preserve a good quality of life (e.g. Hagerty et al., 2005).

## Defining Hope

Although hope is a nearly ever-present phenomenon in human life, defining the construct is difficult. In several scholarly disciplines, many theories and definitions of hope have been proposed (for an overview see Kube et al., 2019; Webb, 2007). According to most definitions, hope involves desiring a future event or outcome with a low or unknown probability of fulfillment (Kube et al., 2019). Although there is a certain overlap between hope and expectations, people can differentiate between these constructs (Montgomery et al., 2003), with expectations relating to subjectively higher certainty of the desired outcome (Kube et al., 2019; Leung et al., 2009). Hope also resembles optimism defined as “generalized expectations of the occurrence of good outcomes in one’s life” (Scheier & Carver, 1985, p. 239) and both involve positive affect towards the future (Bruininks & Malle, 2005). In contrast, pessimism describes anticipating bad outcomes (Scheier & Carver, 1985, p. 219) and is negatively correlated with optimism. However, people distinguish between hope and optimism: Compared to optimism, hope is directed at more important outcomes, with a smaller subjective likelihood of occurring, and less perceived personal control over the obtaining of the outcome (Bruininks & Malle, 2005).

Webb (2007) integrated hope theories and definitions and developed a model with five modes of hoping assigned to the two superordinate dimensions “goal-directed hope” and “open-ended hope”. In a qualitative study, Eaves et al. (2014) applied Webb’s framework to a medical context. Chronic pain patients participating in a randomized-controlled trial (RCT) to evaluate Traditional Chinese Medicine were interviewed before treatment started and over the 18-month course of the RCT. Five modes of hoping emerged from the patients’ answers: *realistic hope*, *wishful hope*, *utopian hope*, *technoscience hope,* and *transcendent hope*. This modes-of-hoping framework will be the theoretical basis of our hope scale.

### Definitions of the Modes of Hoping

Realistic hope describes “any hope that would be considered reasonable or probable based on current medical knowledge” (Eaves et al., 2014, p. 228). It includes, for example, hopes for minor symptom reductions, needing less medication or finding new techniques to manage pain (p. 229). There is a certain overlap between realistic hope and expectations, with realistic hope resembling the definition of the term “hope” in everyday language (e.g. “desire accompanied by expectation of or belief in fulfillment”, Merriam-Webster, n.d.). Wishful hope comprises very high hopes which still can be fulfilled. For example, when patients expressed “hope for a cure” or hope “related to hearsay about miraculous outcomes experienced by others” (Eaves et al., 2014, p. 229). Although patients often considered these hopes to be unrealistic, they are in the realm of possibility and motivate chronically ill patients to seek further treatment. Utopian hope contains hoping that collective action might lead to a better future. In the context of medical and psychological research, utopian hope means that patients hoped that their participation in a research study would contribute to greater overall knowledge about the disease and would help others in the future (Eaves et al., 2014, p. 229). Especially utopian hope and realistic hope show a certain overlap with self-efficacy, i.e., the belief that a certain behavior will produce the desired outcome (i.e., outcome expectancies) combined with the confidence in one’s ability to perform the required behavior, i.e., efficacy expectancies (Bandura, 1977). However, in utopian hope it is a desire rather than an expectancy. Realistic hope also includes outcomes independent from one’s own actions.

Technoscience hope refers to hope for unforeseeable medical or scientific breakthroughs concerning treatment or cure. It also includes faith in science and medicine (Eaves et al., 2014, p. 229). An open, hopeful attitude not directed to a specific outcome or goal is classified as transcendent hope (Eaves et al., 2014, p. 230). Transcendent hope may also contain religious faith and openness to the future.

## Measuring Hope

Due to the variety of definitions, more than 30 measures exist to assess hope (Schrank et al., 2008). They differ in the number of assessed dimensions, whether they assess hope as a trait vs. as a state or globally vs. in a specific context. Although some widely used questionnaires have been developed for clinical settings and used in medical and nursing research (e.g. the Herth Hope Index by Herth, 1992), none of them contains items to assess hope concerning the course of an illness, treatment success or quality of life. Instead, they assess hope more globally, such as having goals or plans for the future, feeling connected to others, and spirituality. Although these questionnaires might be valuable to assess a general hopefulness, possibly linked to positive health outcomes, they do not cover concrete hopes regarding illness or treatment. Additionally, they do not account for hopes concerning participating in a research study. Covering these aspects of hope is the main goal of the newly developed hope scale presented in this article, the Hope in Medicine (HIM) scale.

## Aims of the Present Study

In the present study, we aimed to preliminarily validate the HIM scale by examining its psychometric properties, i.e., its factorial structure, internal consistency and correlations with related constructs such as treatment expectancies, optimism, pessimism, and self-efficacy. We predicted the HIM scale to have an internal consistency of Cronbach’s α ≥ .70. In terms of convergent validity, we predicted a moderate relationship (.3 ≥ *r* ≤ .7) of hope as assessed with the HIM scale with related constructs.

We examined these aspects in a RCT comparing the effects of open-label placebos + treatment as usual (subsequently referred to as “OLP”) vs. treatment as usual (TAU) in allergic rhinitis patients. The main results of this RCT are reported elsewhere (Kube et al., 2022). Here, we focus on the validation of the scale that – in the context of the specific aforementioned RCT – assessed hope concerning the effects of placebo treatment and the course of allergic symptoms. In terms of the modes-of-hoping framework, we assessed hope regarding symptom improvement (realistic hope), hope for full remission of symptoms and/or being cured from allergic rhinitis in the future (wishful hope), and hope that taking part in a research study would contribute to greater knowledge about allergic rhinitis and its treatment (utopian hope). Furthermore, we assessed an open, hopeful attitude towards the future in general (transcendent hope) and hope for unforeseeable scientific breakthroughs concerning novel treatment options for allergic rhinitis (technoscience hope).

In the OLP literature there is a recent discussion whether hope might be a better explanatory mechanism for OLP effects than expectations (e.g. Kaptchuk, 2018). While positive expectations robustly predict effects in deceptive placebos (e.g. Enck et al., 2013), expectations do not predict OLP effects in most studies (e.g. Kleine-Borgmann et al., 2019; Pan et al., 2020). In RCTs, many participants do not report positive treatment expectations; instead, they often report hope (e.g. Eaves et al., 2014; Haas et al., 2022). Therefore, we tested whether hope predicted course of symptoms and quality of life in OLP and TAU to examine criterion validity.

## Materials and Method

### Scale Development

Items were generated by reviewing literature, especially the framework by Eaves et al. (2014, 2016), and by reviewing existing scales. Kube et al. (2019) stated that participating in a research study to evaluate novel treatments could include utopian hope (increasing knowledge), transcendent hope (being open to see what happens), and technoscience hope (hoping for unforeseen medical/scientific breakthroughs). These considerations were also taken into account when developing the items. Reviewing existing questionnaires assessing hope, we included two items (items no. 20, 21) of the Perceived Hope Scale by Krafft et al. (2019) in our questionnaire. Additionally, our scale development was guided by participants’ answers in qualitative studies in which they were asked what they expected or hoped for prior to a new medical treatment (Di Blasi et al., 2005; Eaves et al., 2014, 2015, 2016; Kaptchuk et al., 2009). An initial item-pool of 22 items was developed by one of the authors (LB) in consultation with a second author (TK). Based on the discussion with two further authors (TJK, SKB), who have extensively addressed the concept of hope in both their scientific and clinical work, the wording of six items was slightly revised and five items were replaced entirely. As a result, the HIM scale consisted of 22 items (see Table A1 in Appendix A, Supplementary Materials) that were rated on a 6-point Likert-type scale ranging from 1 = *do not agree* to 6 = *completely agree*. Lower sum scores of the HIM scale indicate less hope. The scale was developed and administered in German (see Table A2 in Appendix A, Supplementary Materials), and it was translated into English for the present article.

### Participants

**Figure 1:**
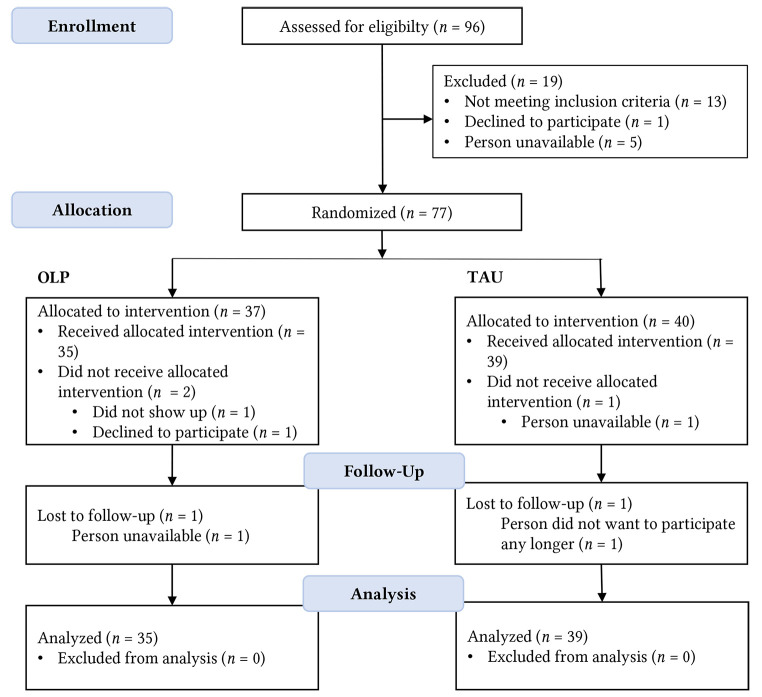
CONSORT Diagram

For the RCT, we aimed to reach a sample size of 90 participants, *f* = .30; α = .05; 1-β = .80, as pre-registered: https://aspredicted.org/ss6ag.pdf (see Kube et al., 2022). 96 participants were screened for study participation. Inclusion criteria were: diagnosed allergic rhinitis, at least 18 years old, and sufficient German language skills. Exclusion criteria were: diabetes, pregnancy, mental or neurological illnesses, and lactose intolerance (as the placebo tablets contained lactose). The final study sample consisted of 74 participants (*n* = 54 female, 73%; *M* = 32.4, *SD* = 13.0 years) as detailed in the CONSORT diagram (see Figure 1). The sociodemographic characteristics are presented for the two treatment conditions separately in Appendix B, Supplementary Materials. Participants were recruited via email lists, social media, and newspaper announcements. Data was collected between April and August 2021. Participants received either 10 € or course credit for their participation.

### Procedure

The RCT included a pretest (t1) and a posttest (t2) with a virtual clinical encounter each time. At t1, a psychology master student spoke to the participants about their allergic rhinitis and informed them about potentially positive effects of placebos. Afterwards, participants completed several questionnaires including the HIM scale. At the end of the pretest (i.e., after completing the questionnaires), participants were informed about their randomized treatment allocation to OLP vs. TAU. In the following 2 weeks, they took either OLP (two placebo tablets per day) + TAU or TAU alone. Participants in the TAU group only took their regular antiallergic medication (if there was any). After 2 weeks, there was a second clinical encounter (t2), in which the same psychology master student asked the participants about the course of their allergic symptoms and potential treatment effects, in addition to the second completion of questionnaires. All data was collected online via the survey platform SoSci Survey (Leiner, 2021). The study was approved by the local ethics committees of the University of Koblenz-Landau and the Johannes Gutenberg University of Mainz. All participants gave informed consent.

### Additional Measures

Severity and frequency of allergic rhinitis symptoms were assessed with the Combined Symptom Medication Score (CSMS; Pfaar et al., 2014) and a questionnaire by Schaefer et al. (2016, 2018). Allergy-related impairment of quality of life was assessed with the German version of the Mini Rhinoconjunctivitis Quality of Life Questionnaire (MiniRQLQ; Juniper et al., 2000). Treatment expectations were measured with the adapted version (Kube et al., 2021) of the Treatment Expectancy Scale by Kube et al. (2020). Self-efficacy, optimism, and pessimism were assessed with the Fragebogen zu Selbstwirksamkeit, Optimismus, Pessimismus Kurzform (SWOP-K9; Questionnaire for Self-Efficacy, Optimism, and Pessimism; Scholler et al., 1999). The instructions were adapted where necessary to refer to the last 2 weeks instead of the last week. These measures are detailed in Appendix C, Supplementary Materials.

### Statistical Analyses

Two participants dropped out between pretest and posttest. Therefore, we conducted an intention to treat analysis with expectation maximization using IBM SPSS Statistics (version 27) to estimate missing values concerning symptom severity, symptom frequency, and quality of life at t2 of those two participants. Power analyses were conducted in G*Power (version 3.1.9.6, Faul et al., 2007), and all other statistical analyses were performed in R (R Core Team, 2020). Alpha error levels were set at 5%.

We conducted an item analysis of the HIM scale and excluded items with a popularity index > 95 according to Dahl (1971; Kelava & Moosbrugger, 2020; see Appendix D, Supplementary Materials). To examine the factorial structure, an EFA was performed with the remaining items. The number of empirically relevant factors was determined with a parallel analysis according to Horn (1965) and an oblique rotation (promax) with these factors was performed as they were expected to be correlated. Items either loading > .30 on more than one factor or not loading at least .30 on any of the extracted factors were excluded (Boateng et al., 2018).

Internal consistency was determined by computing Cronbach’s alpha. To determine the convergent validity, we computed correlations between hope and treatment expectancies, optimism, pessimism, and self-efficacy. Evaluating the criterion validity, three hierarchical regression analyses were conducted to test whether higher hopes at t1 were associated with less symptom severity, frequency of symptoms, and impairment of quality of life after the 2-week intake of OLP or TAU. In the first step, the four hope subscales were included as predictors. In the second step, treatment condition (OLP vs. TAU) was added as an additional predictor. Residuals were plotted to examine whether the preconditions of homoscedasticity, normal distribution of residuals, and correct model specification were met.

## Results

### Item Analysis and Exploratory Factor Analysis

Means, standard deviations, and item popularity according to Dahl (1971) for all items are presented in Appendix E, Supplementary Materials. Items no. 4, 5, 10, and 11 were excluded from further analyses as they had a popularity index > 95. With the remaining 18 items, we performed an EFA. The Kaiser-Meyer-Olkin criterion was .65 and thus above the cutoff of .50 (Kaiser & Rice, 1974) and the Bartlett test was significant (*p* < .001), both suggesting that conducting an EFA is appropriate. A parallel analysis according to Horn (1965) yielded a four-factor solution, explaining 49% of the variance. Table 1 shows the factor loadings and communalities after oblique rotation.

**Table 1:** Results From the Exploratory Factor Analysis of the Hope in Medicine Scale

Item	Factor Loadings					
	1	2	3	4	*h^2^*
Factor 1: realistic hope					
1. I hope I will have less symptoms after taking the pills.	**.93**	-.10	-.10	.19	.86
2. I hope that taking the pills will improve my quality of life.	**.86**	-.05	-.09	.16	.74
3. I have hope that the pills will help me.	**.78**	.06	-.08	.11	.63
7. I believe there is a small chance the placebos will make my symptoms go away completely.	**.39**	.10	.09	-.06	.21
Factor 2: transcendent hope					
21. I am hopeful with regard to my life.	-.02	**.89**	-.05	-.04	.75
19. I have the feeling that a lot of positive things await me in my future life.	-.01	**.85**	.08	.00	.76
18. I have the feeling that my life will develop positively in the future.	.10	**.76**	.00	-.03	.62
20. In my life hope outweighs anxiety.	.05	**.44**	.23	-.17	.31
Factor 3: utopian hope					
8. I have hope that my participation in this study will contribute to a greater overall knowledge about the treatment of allergic rhinitis.	-.29	.07	**.96**	.04	.89
9. I hope that my participation in this study will contribute to helping other people with allergic rhinitis in the future.	-.15	.13	**.82**	-.21	.69
12. I do not believe that I will make an important contribution to the investigation of treatments for allergic rhinitis by participating in this study. (R)	.14	-.09	**.40**	.07	.21
13. I think that studies like this can help us learn more about allergic rhinitis and its treatment.	.03	-.05	**.30**	.26	.18
Factor 4: technoscience hope					
14. I hope that sooner or later an effective treatment for allergic rhinitis will be developed.	-.02	-.04	.21	**.82**	.75
16. I hope for a scientific breakthrough in the treatment of allergic rhinitis.	-.02	-.09	.02	**.70**	.48
17. I have hope that my allergic rhinitis will suddenly be cured someday.	.13	.04	-.13	**.43**	.22
Excluded items					
6. I do not have specific expectations for the treatment with placebos but it is worth trying.	**-.55**	-.01	.10	**.32**	.34
15. I believe science will be able to find a treatment for almost every illness.	-.08	.23	-.12	.17	.09
22. I look hopelessly into the future. (R)	.06	.08	.08	.06	.04

*Note. N* = 74. Extraction method: principal factor analysis with oblique rotation. Factor loadings ≥ .30 are in bold. *h^2^* = communalities. Eigenvalue of factor 1 realistic hope = 3.20; eigenvalue of factor 2 transcendent hope = 1.91; eigenvalue of factor 3 utopian hope = 1.17; eigenvalue of factor 4 technoscience hope = 0.83.

Item no. 6 was excluded from further analyses as it loaded > .30 on more than one factor. Items no. 14 and 22 did not load on any of the four extracted factors, thus they were also excluded. Hence, the final HIM scale contains 15 items comprising four factors: *realistic hope*, *transcendent hope*, *utopian hope*, and *technoscience hope*. The assumed fifth factor *wishful hope* could not be confirmed. Table 2 shows the intercorrelations of these factors.

**Table 2:** Intercorrelations of the Four Factors

Factor	1	2	3	4
1. Realistic hope	–	.24 [-0.11, 0.54]	.18 [-0.16, 0.49]	.23 [-0.11, 0.52]
2. Transcendent hope	.24 [-0.11, 0.54]	–	.29 [-0.06, 0.58]	.09 [-0.21, 0.38]
3. Utopian hope	.18 [-0.16, 0.49]	.29 [-0.06, 0.58]	–	.12 [-0.20, 0.43]
4. Technoscience hope	.23 [-0.11, 0.52]	.09 [-0.21, 0.38]	.12 [-0.20, 0.43]	–

*Note. N* = 74. 95% confidence intervals in square brackets using Bonferroni-Holm correction.

### Internal Consistency and Validity Analyses

Cronbach’s α for the final 15-item HIM scale was .78.

#### Convergent Validity

Table 3 shows the correlations of the four factors of the HIM scale with treatment expectancies, optimism, pessimism, and self-efficacy. Means and standard deviations of the hope subscales and the scales used for validation can be found in Appendix F, Table F1, Supplementary Materials.

**Table 3:** Correlations of the Four Factors of the Hope in Medicine Scale With Treatment Expectancies, Optimism, Pessimism, and Self-Efficacy

Hope Subscales	Treatment Expectancies	Optimism	Pessimism	Self-Efficacy
Realistic hope	.54*** [0.23, 0.75]	.13 [-0.20, 0.44]	-.03 [-0.29, 0.23]	.07 [-0.23, 0.35]
Transcendent hope	.37* [0.03, 0.64]	.50*** [0.18, 0.73]	-.43*** [-0.68, -0.09]	.36* [0.02, 0.63]
Utopian hope	.25 [-0.10, 0.55]	.10 [-0.21, 0.39]	.03 [-0.25, 0.31]	-.01 [-0.24, 0.22]
Technoscience hope	.17 [-0.16, 0.47]	.13 [-0.20, 0.43]	.11 [-0.21, 0.41]	.25 [-0.10, 0.54]

*Note. N* = 74.

*p < .05. **p < .01. ***p < .001. *p* values and 95% confidence intervals in square brackets using Bonferroni-Holm correction.

#### Criterion Validity

Contrary to our assumptions, none of the hope subscales predicted symptom severity (see Appendix F, Table F2, Supplementary Materials) or symptom frequency at t2 (see Appendix F, Table F3, Supplementary Materials). Taking Bonferroni correction into account, none of the hope subscales predicted impairment of quality of life at t2 (see Table 4). The power to detect a small effect of *f^2^* = 0.02 (α = .05, *N* = 74) was very low, though, 1-β = .12.

**Table 4:** Hierarchical Regression Analysis With Impairment of Quality of Life at t2 as Dependent Variable

Predictor	*B*	*SE(B)*	β	*t*	*p*
Model 1					
Realistic hope	-0.08	0.12	-.08	-0.66	.51
Transcendent hope	0.01	0.15	.01	0.09	.93
Utopian hope	0.43	0.18	.30	2.43	.02
Technoscience hope	0.06	0.13	.06	0.49	.62
Model 2					
Realistic hope	-0.07	0.12	-.07	-0.59	.56
Transcendent hope	0.01	0.15	.01	0.09	.93
Utopian hope	0.41	0.18	.28	2.33	.02
Technoscience hope	0.08	0.13	.08	0.64	.52
Treatment condition	0.42	0.23	.21	1.84	.07

*Note.* Model 1: *R*^2^ = .09, *F*(4, 69) = 1.74, *p* = .15. Model 2: *R*^2^ = .14, *F*(1, 68) = 2.12, *p* = .07.

## Discussion

We developed the Hope in Medicine (HIM) scale to assess hope specifically in a medical context and examined its psychometric properties in a 2-week RCT comparing the effects of OLP vs. TAU on symptoms of allergic rhinitis.

An exploratory factor analysis of the HIM scale yielded a four-factor solution with the factors “realistic hope”, “transcendent hope”, “utopian hope”, and “technoscience hope”. Thus, we could extract 4 of the 5 modes of hoping suggested by Eaves et al. (2014, 2016). However, we did not find a fifth factor relating to the mode “wishful hope”. It might be difficult to assess “wishful hope” in allergic rhinitis patients in general as there is a variety of promising treatment options and a wide range of treatment outcomes which can be considered realistic, including full remission. Wishful hope might be more important in more desperate chronically ill patients with a lower likelihood of experiencing full remission. Nonetheless, based on the current data, the HIM scale allows to assess four of the intended modes of hoping that are relevant especially in medical settings and prior to starting a new treatment, speaking to its validity. Furthermore, the scale shows good internal consistency given the heterogeneity of the construct.

Analyses regarding the convergent validity of the HIM scale provided a mixed pattern of results. Realistic hope correlated significantly with treatment expectancies, speaking to its convergent validity as the items assessing realistic hope also relate to desired improvements following placebo treatment which could be considered probable. Transcendent hope was significantly correlated with treatment expectancies, optimism, and self-efficacy and inversely correlated with pessimism, suggesting convergent validity as well. In contrast, the factors utopian hope and technoscience hope did not correlate with any of the assumed related constructs. However, this is not surprising because both utopian hope and technoscience hope are relatively specific aspects of hope. They neither refer to the general attitude towards the future (i.e., optimism, pessimism) nor to specific treatments outcomes (i.e., treatment expectancies). The SWOP-K9 assesses self-efficacy concerning mastering difficulties instead of a general confidence in being able to show a certain behavior to reach a specific outcome (Bandura, 1977). This might explain why utopian hope did not correlate with self-efficacy in the present study, although it shares a certain overlap with self-efficacy defined by Bandura (1977). Future research may examine whether a more substantial association between utopian hope and self-efficacy can be found with other measures of self-efficacy.

None of the hope subscales predicted symptom severity, symptom frequency or impairment of quality of life after the 2-week intake of OLP or TAU, questioning the predictive validity of the HIM scale. However, the statistical power in the present study was low due to the small sample size, possibly explaining the nonsignificant results. It is worth noting, though, that it is unclear so far whether hope is an explanatory mechanism for OLP effects. Only few studies, which show limitations concerning the assessment of hope, have examined the role of hope in OLP RCTs so far (Haas et al., 2022; Kube et al., 2020; Pan et al., 2020). Possibly, measurable hope does not matter in OLP effects or the course of symptoms. Instead, it might mainly instill the motivation to seek treatment.

### Limitations and Future Directions

The present study has several limitations: 1) The same sample was used for developing and validating the HIM scale. Thus, further validation in another independent sample is recommended. 2) Most items showed ceiling effects leading to limited variance which might explain some of the unexpected non-significant results concerning validation. The ceiling effects might be due to social desirability or a self-selection bias with only those patients expressing interest in the study who hoped to benefit from it. Alternatively, giving information about possible positive effects of placebos during the clinical encounter might have instilled hope. 3) The present study focused on allergic rhinitis and baseline allergic symptoms and baseline impairment were rather low. We assume that the HIM scale can be applied to other medical conditions except for life threatening illnesses. However, the external validity of the present RCT is rather limited. Therefore, it would be valuable to examine the HIM scale’s validity in more severe or chronic diseases as hope might be more important in those cases, possibly leading to higher variance, increased explained variance, and higher correlations with measures of convergent validity. In future studies, criterion validity could be addressed by testing whether treatment conditions differ in certain hope subscales after treatment allocation. Content validity could be tested by examining the relation of certain hope subscales and the BIG-5 traits (e.g., transcendent hope with openness). 4) The psychometric properties of the English version of the HIM scale should be tested, and larger sample sizes are recommended for future studies to increase statistical power.

### Conclusions

Since there has been a lack of measures assessing hope specifically in relation to medical treatment and symptom course, the HIM scale may fill this gap as it covers several modes of hoping in the context of starting a new treatment and participating in a research study. As validated in a sample of patients with allergic rhinitis, the scale shows good internal consistency and the preliminary results for its convergent validity are promising. Contrary to our hypotheses, however, hope was not related to greater symptom improvement following treatment. The current study is just a very first step into more systematically investigating the role of hope, which allows only some very cautious conclusions due to the small sample size and some other limitations.

## Supplementary Materials

The Supplementary Materials include the following items:

- **The pre-registration protocol for the study.** The RCT was preregistered at AsPredicted (see Kube et al., 2021). The development and psychometric evaluation of the HIM scale was not preregistered.
- **Online appendices** (see Balthasar et al., 2024)
  - ***Appendix A*.** *Table A1-2.* Table A1 shows the English translation of the instructions and items of the HIM scale. Table A2 shows the German instructions and items of the HIM scale.
  - ***Appendix B*.** *Table B1.* Table B1 shows sociodemographic characteristics of the two treatment groups separately.
  - ***Appendix C.*** Detailed description of additional measures.
  - ***Appendix D.*** Formula to calculate the popularity index according to Dahl (1971).
  - ***Appendix E*.** *Table E1.* Table E1 shows the results of the item analysis.
  - ***Appendix F.*** *Table F1-3.* Table F1 shows means and standard deviations of the hope scales, treatment expectancies, self-efficacy, optimism, and pessimism. Table F2 shows the results of the hierarchical regression analysis with symptom severity as dependent variable. Table F3 shows the results of the hierarchical regression analysis with symptom frequency as dependent variable.

KubeT.
KirschI.
GlombiewskiJ. A.
WitthöftM.
BräscherA.
 (2021). Effects of remotely provided open-label placebos on allergic symptoms
(ID #63631) [Pre-registration protocol]. PsychOpen. https://aspredicted.org/ss6ag.pdf

10.1097/PSY.000000000000111035980787

BalthasarL.
BräscherA.
KaptchukT. J.
BallouS. K.
KubeT.
 (2024). Supplementary materials to "Development and psychometric evaluation of the Hope in Medicine scale"
[Online appendices]. PsychOpen. 10.23668/psycharchives.14058
PMC1130391239119224

## Data Availability

**Open Data:** The information needed to reproduce all of the reported results are not openly accessible. The data is available on request from the authors. **Code:** Code is not openly accessible. **Open Materials:** The information needed to reproduce all of the reported methodology is not openly accessible.
